# Comparing conventional and high sensitivity troponin T measurements in identifying adverse cardiac events in patients admitted to an Asian emergency department chest pain observation unit

**DOI:** 10.1016/j.ijcha.2021.100758

**Published:** 2021-03-25

**Authors:** Ziwei Lin, Swee Han Lim, Qai Ven Yap, Carol Hui Chen Tan, Yiong Huak Chan, Hung Chew Wong, E Shyong Tai, Arthur Mark Richards, Terrance Siang Jin Chua

**Affiliations:** a Emergency Medicine Department, National University Hospital, National University Health System, Singapore; b Department of Emergency Medicine, Singapore General Hospital, Singapore; c Biostatistics Unit, Yong Loo Lin School of Medicine, National University of Singapore, National University Health System, Singapore; d Department of Clinical Pathology, Clinical Biochemistry, Singapore General Hospital, Singapore; e Division of Endocrinology, National University Hospital, National University Health System, Singapore; f Cardiovascular Research Institute, Department of Medicine, Yong Loo Lin School of Medicine, National University of Singapore, Singapore; g Cardiac Department, National University Hospital, National University Health System, Singapore; h Christchurch Heart Institute, Department of Medicine, University of Otago, New Zealand; i Department of Cardiology, National Heart Centre, Singapore

**Keywords:** Acute coronary syndrome, Biomarkers, Chest pain, Major adverse cardiac events, Myocardial infarction, Troponin

## Abstract

**Background:**

High sensitive cardiac troponin assays can be used for prediction of major adverse cardiac events (MACE) in patients with chest pain.

**Methods:**

We included patients with symptoms suggestive of acute coronary syndrome in the emergency department observation unit. We compared the accuracy of conventional troponin T (cTnT) with high sensitive troponin T (hsTnT) at various ranges, as well as the utility of hsTnT and cTnT in prediction of 30-day and 1-year MACE.

**Results:**

1023 patients were included (68.1% male, median age 56 years). There were 2712 hsTnT and cTnT values compared. hsTnT had a higher AUC than cTnT for 30-day and 1-year MACE. The optimal cut-off of 0-hour hsTnT for 30-day (PPV 34%, NPV 96.6%) and 1-year MACE (PPV 40.2%, NPV 94.2%) was 16 ng/L.

For 844 patients who had values for both 0 and 2 h hsTnT, we proposed a rule-out cut-off of 0 and 2 h hsTnT < 16 ng/L (NPV 97.0%, 95%CI 95.5–98.1%) and a rule-in cut-off of 0 and 2 h hsTnT ≥ 26 ng/L (PPV 58.8%, 95%CI 40.7%-75.4%) for 30-day MACE. Negative 0–2 h delta-hsTnT had poor predictive discriminant capabilities on 30-day (PPV 8.2%) and 1-year MACE (PPV 12.3%).

**Conclusion:**

The cut off values of hsTnT used in the 0 and 2-hour algorithm to rule-out (16 ng/L) and rule-in MACE (26 ng/L) are in the range that previous cTnT assays are unable to measure accurately. Risk scores can be used to further improve NPV of the rule-out group. A fall in hsTnT level acutely is not predictive of MACE.

## 1 Introduction

High sensitive cardiac troponin assays have been shown to be superior to conventional troponin assays at predicting major adverse cardiac events (MACE) up to two years after presentation [1], [2]. This may allow better risk stratification of patients presenting to the emergency department (ED) and more effective resource allocation.

Our institution started using high sensitive troponin T (hsTnT) (*Roche Diagnostics, Elecsys Troponin T high sensitive, 99th percentile upper reference limit [URL] 14 ng/L, 10% coefficient of variation [CV] precision 13 ng/L, Limit of detection [LOD] 5 ng/L*) from March 2010. During the initial period of switching from a conventional to high sensitivity troponin assay, clinicians were apprehensive as the positive predictive value (PPV) for MI was only 19% [3]. There was concern that if the cut off value was lowered to the 99th percentile URL; patients may be investigated unnecessarily, posing an additional burden to the healthcare system.

Data regarding hsTnT and conventional troponin T (cTnT) and their utility in predicting long-term MACE in an Asian population is scarce. The prevalence of myocardial infarction (MI) and coronary heart disease vary among different communities. While Asia has traditionally had lower rates of coronary heart disease, urbanization and changes in lifestyle over the years have led to increasing rates of cardiovascular disease in this region [4]. In Singapore, the age-standardized incidence rate of MI has increased significantly from 212.2 per 100,000 population in 2008 to 233.7 per 100,000 population in 2017 [5].

We conducted a head to head comparison between hsTnT and cTnT (*Roche Elesys 4th Generation, 10% CV precision 30 ng/L, LOD 10 ng/L*) in patients admitted to the ED observation unit for chest pain to study the accuracy of cTnT in comparison with hsTnT at various ranges. Our purpose was to understand how to better incorporate hsTnT levels in our ED chest pain algorithm, and to formulate a rule-in and rule-out strategy using 0 and 2 h hsTnT for risk stratification for 30-day and 1-year MACE for patients presenting to the ED with symptoms suggestive of ACS.

## 2 Methodology

This is a prospective observational study (1st May 2010 to 30th April 2013) involving consented patients aged 21 years and above presenting with symptoms suggestive of acute coronary syndrome (ACS) to the ED of Singapore General Hospital, a tertiary care hospital in Singapore, who were admitted to the ED’s observation unit under the department’s chest pain protocol. Exclusion criteria included those with: i) no cardiac troponin data; ii) a 12-lead electrocardiogram (ECG) diagnostic for myocardial ischaemia or MI; iii) presentation with congestive cardiac failure or hypotension associated with chest pain; iv) presentation consistent with unstable angina; v) unequivocal non-cardiac chest pain; vi) concomitant illnesses requiring admission; viii) pregnancy, or ix) end-stage renal failure on dialysis. Patients were recruited using convenience sampling from Monday to Friday, from 0800 to 2100 h, taking into account the availability of research coordinators. This study was approved by the Institutional Review Board at SingHealth, Singapore.

The chest pain protocol targets patients with chest pain who were suitable for further management in the ED observation unit, as decided by the attending physician. As part of the protocol, patients underwent serial ECGs and serum troponin testing at 0, 2, and 7 h after initial ED presentation. Further details of the protocol can be found in Appendix Table A.

At the time of the study, high-sensitive Troponin T (hsTnT) (Roche Diagnostics, Elecsys Troponin T high sensitive) was used in our institution. We defined an abnormal hsTnT as greater than or equal to 30 ng/L, based on a previous study which identified a cut-off of 10 ng/L for cTnT giving a NPV of 100% for MI [6]. This upper limit of hsTnT was also used in Sweden from 2010 to 2013, as well as in Australia [7], [8]. For this study, additional blood was collected at 0, 2 and 7 h from the time of the first blood draw by the ED doctor. The additional blood was centrifuged on-site and stored at −80 degrees Celsius until utilized for the measurement of conventional troponin T (*Roche Elesys 4th Generation*).

While in the observation unit, patients who developed symptoms consistent with myocardial ischaemia, had dynamic ECG changes, or elevated troponin, were admitted to the inpatient cardiology service. Patients at intermediate risk, as determined by the attending physician, underwent stress nuclear myocardial perfusion imaging within three days. A standardized data set was collected by research staff on each participant, which included demographic variables such as age and gender, past medical history, current medications, presenting signs and symptoms, test results, interventions, and outcomes. Patients were followed up at 30 days and one year via telephone and/or through assessing medical records for outpatient clinic visits (specialist and primary care), emergency department visits, or hospital admissions. Patients were only considered to be followed-up if there were reviews by physicians documented in the electronic records. Electronic healthcare records were also linked to the national death registry. If we were unable to satisfactorily ascertain whether the patient had MACE through electronic healthcare records, they were then contacted by telephone.

The primary outcome was 30-day MACE, and the secondary outcome was 1-year MACE. MACE was defined as occurrence of any of the following: cardiac death, ventricular fibrillation, type 1 myocardial infarction, critical stenosis found on coronary angiogram (≥50% for left main coronary artery stenosis or ≥70% for epicardial vessel stenosis), emergency cardiac revascularisation procedures e.g. coronary artery bypass graft (CABG) or percutaneous coronary intervention (PCI).

The 30-day and 1-year diagnoses and outcomes were independently adjudicated by an emergency medicine attending physician (SHL) and an attending cardiologist (TSJC) based on case records, which included clinical data such as history, physical examination findings, co-morbidities, investigation results and data on troponin collected during the index visit and up to one year of follow-up. Where inter-reviewer differences occurred, discussion was held between the two reviewers to reach consensus.

### 2.1 Statistical analysis

Statistical analyses were carried out using IBM SPSS Statistics 26. Descriptive statistics for numerical variables were presented as mean (SD) when normality assumption was satisfied, and otherwise presented as median (IQR). Categorical variables were presented as n (%). Pearson correlation and linear regression were performed to investigate the relationship between hsTnT and cTnT. Receiver Operating Characteristic (ROC) curves were used to determine predictive capabilities of both cTnT and hsTnT levels at 0, 2, 7 h and delta 0–2 hsTnT. Rule-in and rule-out cut-offs were proposed based on PPV and NPV of 0 and 2 h hsTnT. Various risk scores such as the HEART score, TIMI score, and EDACS were applied to the low-risk category to further risk stratify these patients.

## 3 Results

The study population consisted of 1023 patients with 68.1% male, median age of 56 years (interquartile range [IQR] 48 to 63 years, Appendix Table B). Majority (n = 913, 89.2%) had a chief presenting complaint of chest pain, 245 (23.9%) had a history of ischaemic heart disease or coronary artery disease and 92 (9.0%) had previous MI. The median time from onset of symptoms to first troponin done was 286 min (IQR 160 to 715.5 min). For outcomes, 68 (6.6%) had 30-day MACE (inclusive of index MACE) and 96 (9.4%) had 1-year MACE (inclusive of index and 30-day MACE).

### 3.1 hsTnT and correlation with cTnT values

**Fig. 1.1:**
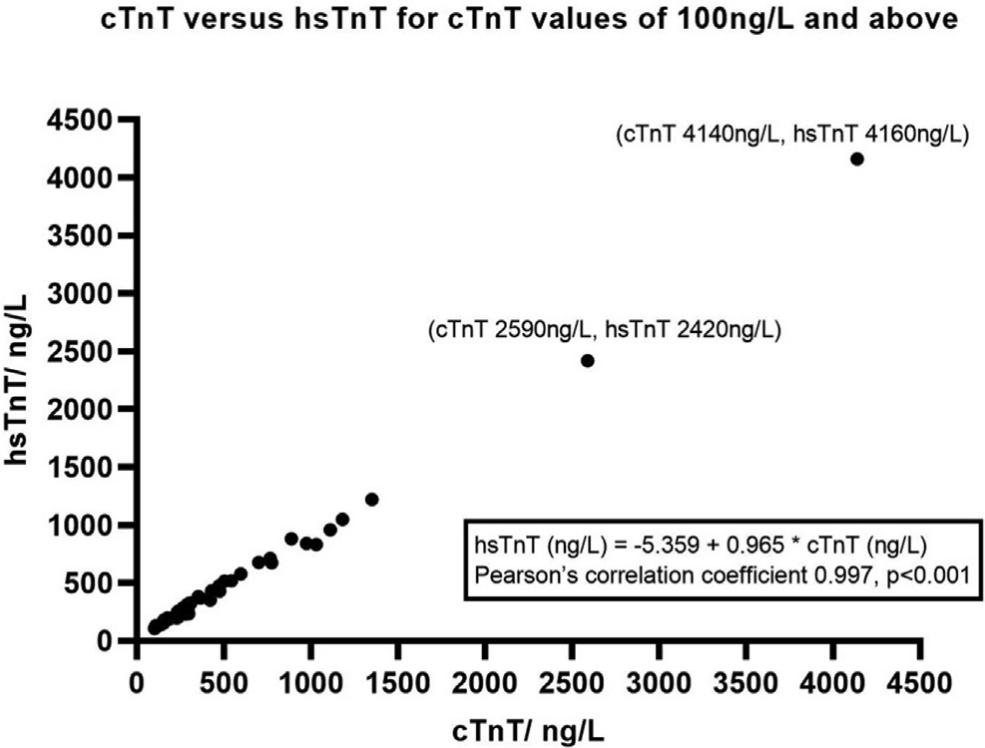
cTnT versus hsTnT for cTnT values of 100 ng/L and above.

**Fig. 1.2:**
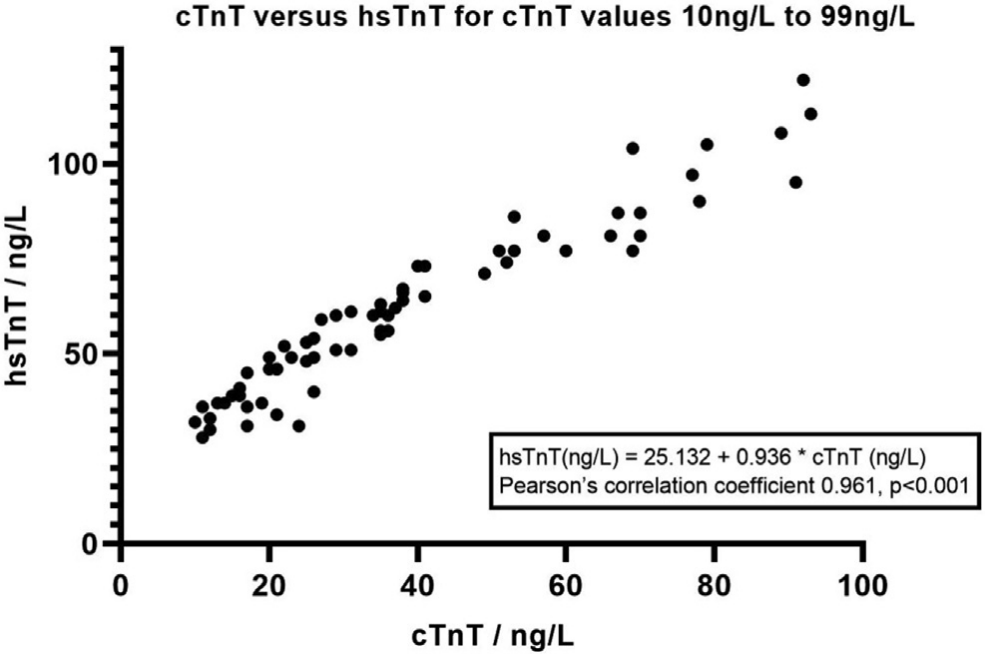
cTnT versus hsTnT for cTnT values of 10 ng/L to 99 ng/L.

A total of 2712 hsTnT and cTnT values were compared against each other regardless of the time at which they were taken (0, 2, or 7 h). cTnT values were categorized into<10 ng/L (the smallest value of cTnT reported), 10 ng/L to 99 ng/L, and 100 ng/L and above in order to study the correlation between cTnT and hsTnT values (Fig. 1.1, Fig. 1.2).

cTnT values of 100 ng/L and above (range 100 ng/L to 4140 ng/L, n = 49) corresponded to hsTnT of 106 ng/L to 4160 ng/L (r = 0.997, p < 0.001). For cTnT values 10 ng/L to 99 ng/L (n = 67), the corresponding range of hsTnT is 28 ng/L to 122 ng/L (r = 0.961, p < 0.001). We derived a correlation formula of hsTnT = 25.132 + 0.936*cTnT. The correlation coefficient of cTnT values of 100 ng/L and above versus the corresponding hsTnT values is higher than that of cTnT values of 10 ng/L to 99 ng/L (p < 0.05).

cTnT < 10 ng/L (n = 2596) corresponded to a wide spread of values for hsTnT (range < 3 ng/L to 42 ng/L, median 5 ng/L IQR < 3 ng/L to 7 ng/L). There were 1010 readings (38.9%) with hsTnT value of < 3 ng/L and 18 readings (0.69%) of hsTnT ≥ 28 ng/L (corresponding value of cTnT 10 ng/L [9]).

### 3.2 hsTnT versus cTnT in predicting 30-day and 1-year MACE

**Fig. 2:**
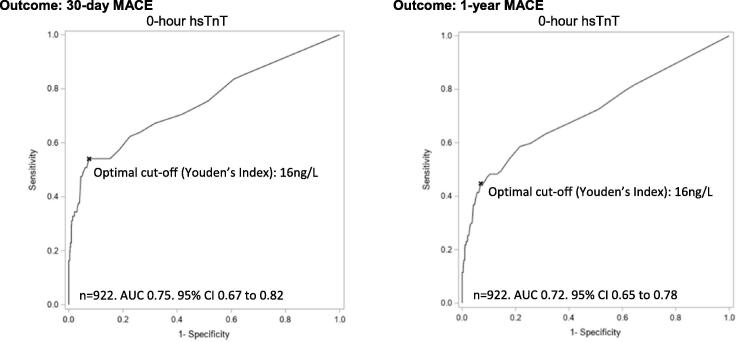
ROC curves for 0-hour hsTnT cut-offs versus 30-day and 1-year MACE.

The c-statistics (AUC) of hsTnT were consistently higher than cTnT for 30-day and 1-year MACE (Table 1) with an optimal cut-off level (determined using Youden Index) of 0-hour hsTnT for 30-day and 1-year MACE of 16 ng/L (Fig. 2).

**Table 1:** hsTnT and cTnT and association with 30-day and 1-year MACE.

Troponin used (N)	AUC, 95% CI	Optimal cut-off by Youden’s Index	Sensitivity/%	Specificity/%	PPV/%	NPV/%
Outcome: 30-day MACE						
0-hour cTnT (9 2 2)	0.66, 0.60 to 0.72					
2-hour cTnT (9 2 9)	0.70, 0.63 to 0.76					
7-hour cTnT (8 6 2)	0.72, 0.66 to 0.79					
0-hour hsTnT (9 2 2)	0.75, 0.67 to 0.82	≥16 ng/L	54.1	92.6	34.0	96.6
2-hour hsTnT (9 2 9)	0.75, 0.67 to 0.84	≥13 ng/L	62.3	89.9	30.2	97.1
7-hour hsTnT (8 6 2)	0.75, 0.66 to 0.84	≥16 ng/L	60.3	93.4	39.8	97.0
Outcome: 1-year MACE						
0-hour cTnT (9 2 2)	0.61, 0.56 to 0.65					
2-hour cTnT (9 2 9)	0.64, 0.59 to 0.69					
7-hour cTnT (8 6 2)	0.66, 0.61 to 0.72					
0-hour hsTnT (9 2 2)	0.72, 0.65 to 0.78	≥16 ng/L	44.8	93.1	40.2	94.2
2-hour hsTnT (9 2 9)	0.71, 0.64 to 0.78	≥11 ng/L	55.2	86.8	30.2	94.9
7-hour hsTnT (8 6 2)	0.70, 0.63 to 0.78	≥11 ng/L	54.8	86.3	28.0	95.1

There is a positive linear relationship between the level of hsTnT and PPV of MACE (Table 2). The 0, 2, and 7-hour hsTnT plateaued at ≥ 90 ng/L with a PPV of > 80% for both 30-day MACE (Fig. 3.1) and 1-year MACE (Fig. 3.2). The 2 and 7-hour hsTnT cut-offs ≥ 35 ng/L correspond to PPV > 60%.

**Table 2:** hsTnT cut-offs at 0 and 2 h for 30-day and 1-year MACE.

	MACE at 30 days				MACE at 1 year			
0-hour hsTnT / ng/L (≥*x*)	Sensitivity/%	Specificity/%	PPV/%	NPV/%	Sensitivity/%	Specificity/%	PPV/%	NPV/%
5	75.4	48.6	9.4	96.5	72.4	49.0	12.9	94.5
6	70.5	58.2	10.6	96.5	67.8	58.8	14.6	94.6
12	54.1	86.3	21.9	96.4	48.3	87.0	27.8	94.2
13	54.1	88.9	25.6	96.5	48.3	89.6	32.6	94.3
14	54.1	90.0	27.7	96.5	47.1	90.7	34.5	94.3
15	54.1	91.5	31.1	96.6	44.8	92.0	36.8	94.1
16	54.1	92.6	34.0	96.6	44.8	93.1	40.2	94.2
26	34.4	97.5	48.8	95.5	25.3	97.5	51.2	92.6
30	32.8	97.9	52.6	95.4	24.1	98.0	55.3	92.6
35	32.8	98.5	60.6	95.4	23.0	98.4	60.6	92.5
52	26.2	99.1	66.7	95.0	18.4	99.0	66.7	92.1
90	19.7	99.7	80.0	94.6	13.8	99.6	80.0	91.7
**2-hour hsTnT / ng/L (≥*x*)**	**Sensitivity/%**	**Specificity/%**	**PPV/%**	**NPV/%**	**Sensitivity/%**	**Specificity/%**	**PPV/%**	**NPV/%**
5	75.4	48.6	9.3	96.6	70.1	48.8	12.4	94.1
6	72.1	58.4	10.8	96.8	66.7	58.8	14.3	94.5
12	62.3	88.9	28.1	97.1	51.7	89.3	33.3	94.7
13	62.3	89.9	30.2	97.1	50.6	90.3	34.9	94.7
14	60.7	90.9	31.9	97.1	49.4	91.4	37.1	94.6
15	59.0	92.5	35.6	97.0	46.0	92.8	39.6	94.3
16	57.4	93.2	37.2	96.9	44.8	93.5	41.5	94.3
26	45.9	97.5	56.0	96.3	33.3	97.5	58.0	93.4
30	44.3	97.7	57.4	96.2	31.0	97.6	57.4	93.2
35	39.3	98.2	60.0	95.8	27.6	98.1	60.0	92.9
52	36.1	99.0	71.0	95.7	25.3	98.9	71.0	92.8
90	24.6	99.7	83.3	95.0	17.2	99.6	83.3	92.1

**Fig. 31:**
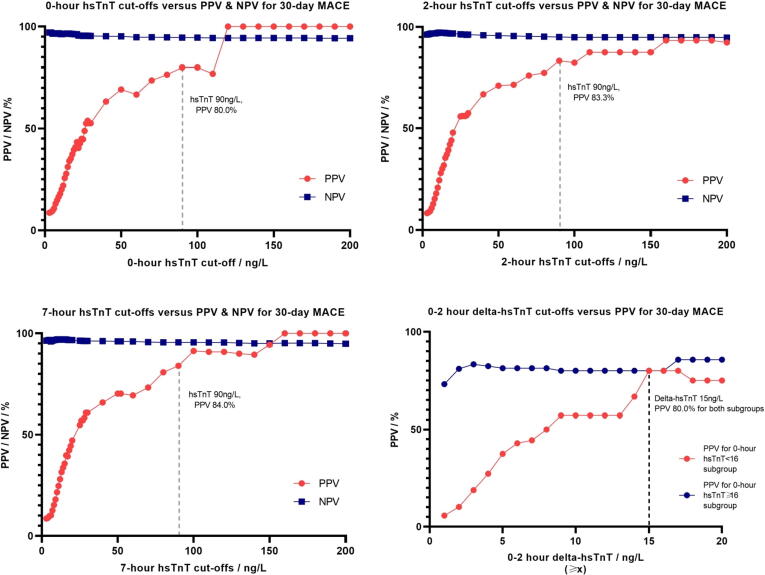
Trend of PPV and NPV for 0, 2, and 7 h hsTnT and 0–2 h delta-hsTnT cut-offs for 30-day MACE.

**Fig. 32:**
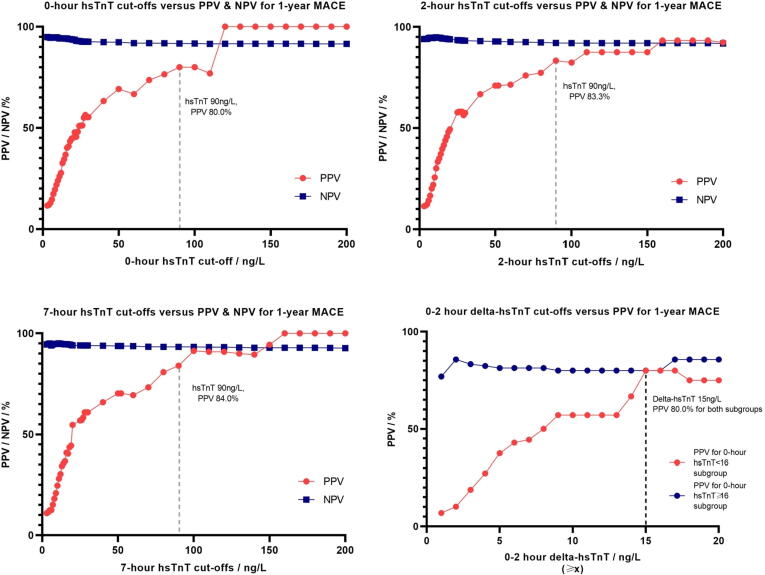
Trend of PPV and NPV for 0, 2, and 7 h hsTnT and 0–2 h delta-hsTnT cut-offs for 1-year MACE.

### 3.3 Absolute delta-hsTnT in predicting 30-day MACE and 1-year MACE

There were 844 (82.5%) with both 0-hour and 2-hour hsTnT, and 201 (23.8%) with positive 0–2 h delta-hsTnT. A positive 0–2 h delta-hsTnT gave an AUC of 0.83 (95% CI 0.73 to 0.94) for 30-day MACE and AUC 0.80 (95% CI 0.70 to 0.91) for 1-year MACE. For those with 0-hour hsTnT ≥ 16 ng/L, any increase in 2-hour hsTnT led to a PPV of > 70% for 30-day MACE. However, for 0-hour hsTnT < 16 ng/L, the PPV for 30-day MACE only increased to 50% when the 0–2 h delta-hsTnT was ≥ 8 ng/L (Fig. 31, Fig. 32).

There were 220 patients (26.1%) with negative 0–2 h delta hsTnT. Negative 0–2 h delta-hsTnT had poor predictive discriminant capabilities on MACE (specificity 74.3%, PPV 8.2% for 30-day MACE; specificity 74.7%, PPV 12.3% for 1-year MACE). In this group, a 2-hour hsTnT below 26 (n = 202) had a PPV 5.9% and specificity 5.9%; and PPV 10.4% and specificity 6.2% in predicting 30-day and 1-year MACE respectively.

### 3.4 Rule-in and rule-out cut-offs for 30-day MACE

**Fig. 4:**
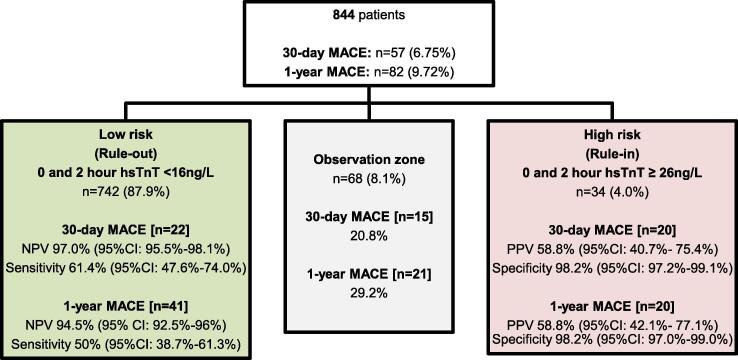
Proposed rule-in and rule-out cut-offs for 30-day and 1-year MACE.

A rule-out cut-off of 0 and 2 h hsTnT < 16 ng/L (rule-out group) and a rule-in cut-off of ≥ 26 ng/L (rule-in group) (Fig. 4) was proposed. Fifty-seven (6.75%) had 30-day MACE, including 34 (4.0%) with MI, and no deaths. Eighty-two (9.72%) had 1-year MACE, which included 40 (4.7%) with MI, and 1 (0.1%) cardiac related mortality. The only patient with 1-year cardiac related mortality was accurately classified in the rule-in zone.

There were 14 patients in the rule-in group (0 and 2 h hsTnT ≥ 26 ng/L) who did not have 1-year MACE. In these 14 patients, the mean serum creatinine was 279.2 μmol/L (standard deviation [SD] 211.4 μmol/L), which was higher than that of the combined rule-in group (mean 82.2 μmol/L, SD 48.5 μmol/L). Eleven (78.6%) had previous MI or known coronary artery disease, and of the remaining three, one of them had undiagnosed hypertrophic obstructive cardiomyopathy.

In the rule-in group, patients with an initial 0-hour hsTnT of ≥ 26 ng/L and a positive 0–2 h delta-hsTnT had a PPV of 92.9% for 30-day MACE.

#### 3.4.1 Utility of 7-hour hsTnT

Further analysis was done to evaluate the utility of 7-hour hsTnT in predicting MACE. Among 844 patients with both 0 and 2 h hsTnT, 837 had 7-hour hsTnT readings. The seven who did not have 7-hour hsTnT readings were in the rule-out group. When the 7-hour hsTnT cut-off of ≥ 26 ng/L was applied to these patients, the sensitivity, specificity, PPV, and NPV was 53.6%, 96.8%, 54.5%, and 96.7% for 30-day MACE; and 40.0%, 97.0%, 58.2%, and 93.9% for 1-year MACE respectively.

Among those who met the rule-out cut-offs (0 and 2-hour hsTnT < 16 ng/L) and who had 7-hour hsTnT readings (n = 735), 21 (2.9%) had 30-day MACE, and only 2 (9.5%) out of the 21 had a 7-hour hsTnT reading of ≥ 26 ng/L. A total of 39 (5.3%) out of the 735 patients had 1-year MACE, out of whom 2 (5.1%) had 7-hour hsTnT of ≥ 26 ng/L.

There were 15 patients with 30-day MACE in the observation zone, and among them 8 (53.3%) had a 7-hour hsTnT ≥ 26 ng/L. A total of 21 patients in the observation zone had 1-year MACE, out of whom 10 (47.6%) had a 7-hour hsTnT ≥ 26 ng/L.

For patients with MACE in the rule-in group (n = 20), all (100%) had 7-hour hsTnT ≥ 26 ng/L. There was significant difference in the 7-hour hsTnT values for those with MACE and without MACE in the rule-in group. The mean for 7-hour hsTnT in the rule-in group for those with no MACE was 60 ng/L (standard deviation [SD] 31 ng/L) versus 1107 ng/L (SD 2240 ng/L) for those with MACE (p < 0.001).

#### 3.4.2 Risk factors for 30-day and 1-year MACE

The HEART score [10], TIMI score [11], and EDACS [12] were applied to the rule-out group (low-risk category) to further risk stratify these patients (Table 3). The cut-off for normal hsTnT was taken to be 16 ng/L as per our low-risk classification. All three scores had similar C-statistics for 30-day and 1-year MACE.

**Table 3:** Risk scores applied to low risk (rule-out) subgroup for 30-day MACE.

Score	AUC (95% CI)	Cut-off used	True positive	False positive	False negative	True negative	Sensitivity/%	Specificity/%	PPV/%	NPV/%
*Outcome: 30-day MACE*										

EDACS	0.73(0.63–0.83)	Low risk* versus not low risk	14	236	8	484	63.6	67.2	5.6	98.4
HEART	0.73 (0.62–0.83)	>2	21	530	1	190	95.5	26.4	3.8	99.5
		>3	16	292	6	428	72.7	59.4	5.2	98.6
		>4	10	103	12	617	45.5	85.7	8.8	98.1
		>5	2	22	20	698	9.1	96.9	8.3	97.2
		>6	1	4	21	716	4.5	99.4	20.0	97.2
TIMI	0.78 (0.69–0.88)	>0	20	439	2	281	90.9	39.0	4.4	99.3
		>1	18	216	4	504	81.8	70.0	7.7	99.2
		>2	11	105	11	615	50.0	85.4	9.5	98.2
		>3	4	26	18	694	18.2	96.4	13.3	97.5
		>4	1	4	21	716	4.5	99.4	20.0	97.2
*Outcome: 1-year MACE*										

EDACS	0.66(0.58–0.75)	Low risk* versus not low risk	24	226	17	475	58.5	67.8	9.6	96.5
HEART	0.73 (0.66–0.80)	>2	40	511	1	190	97.6	27.1	7.3	99.5
		>3	32	276	9	425	78.0	60.6	10.4	97.9
		>4	14	99	27	602	34.1	85.9	12.4	95.7
		>5	3	21	38	680	7.3	97.0	12.5	94.7
		>6	2	3	39	698	4.9	99.6	40.0	94.7
TIMI	0.72 (0.64–0.80)	>0	35	424	6	277	85.4	39.5	7.6	97.9
		>1	28	206	13	495	68.3	70.6	12.0	97.4
		>2	16	100	25	601	39.0	85.7	13.8	96.0
		>3	6	24	35	677	14.6	96.6	20.0	95.1
		>4	1	4	40	697	2.4	99.4	20.0	94.6

* - Low risk patients were considered as those with an EDACS < 16, ECG showing no new ischemia and negative 0 and 2 h troponin results. Patients were not considered to be low risk as long as they did not fulfil one of the criteria.

## 4 Discussion

This study aimed to explore the relationship between cTnT and hsTnT, and found that while there is good correlation for larger values of cTnT, there was poorer correlation for smaller values of cTnT. hsTnT was also found to be a better predictor of 30-day and 1-year MACE as compared to cTnT. The high sensitivity assay was able to accurately detect values of troponin below the LOD of the conventional assay. We also proposed a rule-in and rule-out cut-off for 0 and 2-hour hsTnT to allow patients presenting to the ED with symptoms suggestive of ACS to be risk stratified for 30-day and 1-year MACE. The NPV of our suggested cut-offs can be further improved with the use of established risk scores (e.g. HEART score, TIMI score or EDACS). Of note, the values of the cut-offs that we have selected (16 ng/L for rule-out and 26 ng/L for rule-in) would have fallen below the LOD for the corresponding values of cTnT, and thus may not be possible with conventional assays.

### 4.1 Correlation between hsTnT and cTnT values

The 4th generation cardiac troponin assays have a 10% CV of 30 ng/L [13] (equivalent to hsTnT 49 ng/L) [9] whereas hsTnT assays have a 10% CV of 13 ng/L [14], [15]. Both assays target the same epitopes for capture and detection, however the high-sensitivity assay achieves a lower limit of detection by increasing the concentration of capture and detection antibodies, increased sample volume from 15 μl to 50 μl [16], and buffer optimization to reduce background signal.

Saenger et al had previously shown good correlation between cTnT and hsTnT values (hsTnT = 1.02*cTnT + 18.4, r = 0.99), but noted that there were significant differences at the critical low end of the analytical measuring range when the hsTnT values were < 50 ng/L (corresponding to a cTnT value at its 10% CV of 30 ng/L) [9]. Our study also showed that the Pearson’s coefficient for hsTnT versus cTnT was found to be lower when cTnT values were lower (r = 0.997 for cTnT ≥ 100 ng/L versus r = 0.961 for cTnT 10 ng/L to 99 ng/L). cTnT values of < 10 ng/L (minimum value recorded) had a wide range of hsTnT values. The co-relation formula derived from our study is hsTnT = 25.132 + 0.936*cTnT for cTnT values of 10 ng/L to 99 ng/L. While Saenger et al’s formula gave a value of hsTnT of 49 ng/L for a cTnT of 30 ng/L (10% CV of cTnT); our formula gave a corresponding hsTnT value of 53 ng/L. The difference is likely due to variations in the machines and reagents used to run the tests.

Clinicians may not be aware that samples with troponin T above the concentration of ≥10% CV of cTnT will yield different numerical results when analysed with the cTnT assay as compared to the hsTnT assay. This difference is highly significant in comparing previous cTnT with current hsTnT values whether in the context of clinical work or conducting or interpreting research.

### 4.2 Cut-offs of hsTnT for prediction of long-term MACE

Information regarding specific cut-offs of hsTnT associated with long-term MACE is still lacking. The elevation of cardiac troponin levels above the 99th percentile of a reference control group (or the upper reference limit [URL]) is used to define MI [17]. However, the 99th percentile for each population is different, and there is no standardized guideline as to how this value can be achieved.

While the 99% URL of hsTnT was taken to be 14 ng/L when it was first introduced in Europe [3], other studies have reported varying values from 13 ng/L to 28 ng/L [18], [19]. The optimal cut-off in our study population for hsTnT at 0 h to predict both 30 days and one year MACE is 16 ng/L(Table 1). This value is similar to the 99% URL of hsTnT reported in Singapore (15.2 ng/L) [20]. Substituting 15 ng/L as the cut-off value for 0 and 2 h hsTnT instead of 16 ng/L identified the same number of true positives for 30-day and 1-year MACE, but also inaccurately categorized 9 more false positives for 30-day and 1-year MACE as compared to the 16 ng/L cut-off (Table 2).

Our study showed a positive linear relationship between the level of hsTnT and PPV of MACE (Appendix Fig. 31, Fig. 32). White first suggested an algorithm using an initial and 9-hour hsTnT cut off of < 14 ng/L to rule-out myocardial infarction, whilst an initial hsTnT level of ≥ 53 ng/L and a 3-hour hsTnT with an increase of ≥ 20% will rule-in myocardial infarction [21]. In our study, the cut-off values for 0 and 2 h hsTnT were 16 ng/L for the rule-out zone and 26 ng/L for the rule-in zone (Fig. 4). Other clinical studies have followed a similar concept incorporating hsTnT baseline values and absolute change within the first hours to allow for a safe rule-out and accurate rule-in of myocardial infarction within two hours [22], [23]. Reichlin et al used a rule-out cut-off of 0 and 2 h hsTnT < 14 ng/L and delta 0–2 h hsTnT < 4 ng/L (sensitivity 96%, NPV 99.5% in the validation cohort) and rule-in cut-off of 0 and 2 h hsTnT ≥ 53 ng/L or delta 0–2 h ≥ 10 ng/L (specificity 99%, PPV 85% in the validation cohort) for the diagnosis of acute myocardial infarction [22]. The varying optimal cut-off values between studies despite using the same assay may be related to the different prevalence of ACS and MACE in the different populations. This may also be due to differences in study methodology, such as differences in study outcomes (e.g. diagnosis of acute myocardial infarction versus MACE), and differences in criteria for patient selection.

Very low levels of hsTnT are associated with reduced occurrence of 30-day MACE in patients presenting to the ED with chest pain. In one center, for patients presenting with chest pain and non-ischaemic ECG changes, a cut-off of hsTnT<5 ng/L taken at least 3 h from symptoms onset was able to rule out 30-day MACE (defined as AMI, revascularization, or cardiac death) (NPV 99.0%) [24]. Another study used a cut-off of 0- and 3-hour hsTnT ≤ 19 ng/L for 30-day MACE (NPV 99.3%), and found that lowering the cut-off to 6 ng/L did not increase the NPV [25].

The threshold of hsTnT < 5 ng/L at 0-hours in our cohort of 1023 patients only gave a NPV 96.5% and 94.5% for 30-day and 1-year MACE respectively (Table 2). This may be as patients with symptoms < 3 h from presentation were not excluded, and also due to the different prevalence of MACE in our population. For the 844 patients in our study with both 0- and 2-hour hsTnT values, 0- and 2-hour hsTnT < 5 ng/L gave a NPV of 97.4% and 95.1% for 30-day and 1-year MACE respectively. The NPV was similar to that of 0 and 2-hour hsTnT < 16 ng/L, but had a much higher false positive rate (450/787 [57.2%] compared to 67/787 [8.5%] for 30-day MACE and 433/762 [56.8%] compared to 61/762 [8.0%] for 1-year MACE).

We also found that for patients with the rule-out cut-off of 0 and 2 h hsTnT < 16 ng/L, an additional hsTnT at 7 h is not useful in identifying additional patients with MACE. However 7-hour hsTnT should continue to be performed in patients in the observation and rule-in zone. The cut-off of 7-hour hsTnT ≥ 26 ng/L however may not be appropriate for ruling out MACE as the sensitivity was low for both 30-day (53.6%) and 1-year (40.0%) MACE.

The NPV of 97% for 30-day MACE and 94.5% for 1-year MACE for the rule-out group was improved to above 97% if either the TIMI score, HEART score or EDACS was applied as well (Table 3). Routine follow up at specialist clinics or stress ECG, imaging or CT coronary angiogram may not be cost-effective in this group. Further studies on a larger group are needed to calibrate the use of risk scores for our population.

### 4.3 The role of delta-hsTnT

Changes in serial hsTnT values or the delta value has been used in early diagnosis of MI and in rapid “rule-out” strategies in the ED [26]. The first to fourth universal definition of acute MI includes a fall in cardiac troponin as one of the criteria [17], [27]. There is no literature on the exact magnitude of fall in troponin levels for the diagnosis of MI or risk stratification of acute chest pain patients. Our study has illustrated that a fall in troponin level acutely (within 7 h) is not predictive of 30-day or 1-year MACE.

While absolute delta-hsTnT values are useful in early MI diagnosis [28], there is less evidence regarding their utility for prognosis of long-term outcomes. Absolute and relative delta-hsTnT were previously found to be less useful in prognosticating 1-year MACE as compared to maximum hsTnT in patients presenting with acute chest pain without ECG changes of ST-elevation [29]. In our study, positive delta-hsTnT has shown to be less accurate in prognosticating long-term MACE as compared to absolute hsTnT values in the subgroup with 0-hour hsTnT < 16 ng/L. Hence we proposed a rule-out cut-off of 0 and 2 h hsTnT < 16 ng/L and a rule-in cut-off of 0 and 2 h hsTnT ≥ 26 ng/L as illustrated in Fig. 4.

## 5 Limitations

This study was done in a single center and only among patients admitted to the study site’s ED observation unit. Patients in this group were deemed to be of moderate for MACE by their attending emergency physician. Results hence may not be generalizable to the general population and with all patients with undifferentiated chest pain presenting to the emergency department. However, high risk patients would likely be admitted and undergo cardiac work-up, and there may not be a need to risk stratify them. This makes the results of our study more applicable to a population of patients where risk stratification is necessary in order to utilize resources in a more efficient way.

In addition, patients were followed up at 30 days and 1 year either via telephone or by tracing electronic medical records. More than 60% of the patients were followed up by tracing electronic health records for MACE, while the rest were contacted via telephone. The electronic healthcare records are comprehensive and are able to capture events and encounters that occur not only within the same hospital but also affiliated (i.e. within the same healthcare network) hospitals, specialist centers (e.g. oncology center, heart center, etc.) and primary care clinics. However, events may still have been missed if patients presented to other centers not included in the same healthcare network. We circumvented this by only considering patients to be followed-up if there were reviews by physicians documented in the electronic records. If we were unable to satisfactorily ascertain whether the patient had MACE through electronic healthcare records, they were then contacted by telephone.

## 6 Conclusion

In patients presenting to the ED with symptoms suggestive of ACS, hsTnT taken at 0 and 2 h after presentation may have a role in prognosticating long-term MACE, even up to 1 year after their index visit. hsTnT was also found to be a better predictor of 30-day and 1-year MACE as compared to cTnT. Of note, the cut off values of hsTnT used in our 0 and 2 h algorithm to determine rule-out (16 ng/L) and rule-in (26 ng/L) in the study are in the range that previous conventional troponin assays were unable to measure correctly.

In addition, the acute fall of hsTnT (within 7 h) is not predictive of MACE. Risk scores can be used in conjunction with the 0 and 2 h hsTnT algorithm to further improve NPV of the rule-out group.

As different communities have different ACS prevalence, it may be useful for healthcare institutions to perform follow-up studies in their own respective sites after implementing high sensitivity troponin assays to calibrate cut-off values to rule in and rule out ACS based on prevalence of ACS and resources available.

## Declaration of Competing Interest

Dr. Arthur M. Richards has received, in kind, support from the industry: assay provision by Roche Diagnostics and Abbott Laboratories; research grant from Roche Diagnostics; and speaker’s and advisory board fees from Roche Diagnostics and Novartis.

## Figures and Tables

**Table A:** Emergency department observation unit chest pain protocol.

*Inclusion criteria*	• Patients presenting to emergency department with chest pain suggestive of stable angina or angina equivalent • Initial 12-lead electrocardiogram (ECG) **non-diagnostic** for myocardial ischaemia and acute myocardial infarction
*Exclusion criteria*	• Presence of congestive cardiac failure or hypotension associated with chest pain • A clinical syndrome of persistent chest pain consistent with unstable angina. This includes patients with past history of proven coronary artery disease, and present chest pain is more severe or frequent than previous angina episodes • Definite non-cardiac chest pain • Patient has concomitant illnesses that require admission • Clinical diagnosis of aortic dissection or pulmonary embolism • Paediatric patients and patients from community hospitals
*Investigations done in the emergency department prior to observation ward admission*	• Point-of-care test: ECG (including tracing of old ECGs) • Haematological and biochemical investigations: Full blood count, urea/electrolytes/creatinine, troponin T • Radiological investigations: Chest x-ray
*Interventions*	• Continuous ECG monitoring • Vital signs 2-hourly • Repeat troponin T and ECG at 2 and 7 h • Symptomatic treatment e.g. analgesia, antacid (if for suspected gastroesophageal reflux disease)
*Discharge criteria*	• Negative 8-hour observation • Telemetry review by doctor and no arrhythmias or ventricular tachycardia noted • Reviewed by attending doctor to have low risk of acute coronary syndrome
*Admission criteria*	• Patients who develop recurring chest pain consistent with myocardial ischaemia • Patients who develop cardiac failure or hypotension • ECG changes of ST segment elevation or depression or deep T inversions in leads with dominant R waves • Positive troponin T > 30 ng/L, or > 13 ng/L but rising • For cardiology review for high dependency admission if suggestive of myocardial infarction
*Discharge interventions*	• For stress myocardial perfusion imaging on discharge with cardiology follow-up if deemed appropriate by attending physician

**Table B:** Characteristics of the study population.

Characteristic	Median (IQR) or n (%)
Age	56 (48–63)
**Gender**	
Male	697 (68.1)
Female	326 (31.9)
**Race**	
Chinese	660 (64.5)
Malay	121 (11.8)
Indian	198 (19.4)
Others	44 (4.3)
**Co-morbidities**	
Hypertension	565 (55.2)
Diabetes Mellitus	294 (28.7)
Insulin therapy	32 (3.1)
History of smoking	267 (26.1)
Hyperlipidaemia	543 (53.1)
Family history of IHD/CAD	122 (11.9)
Previous myocardial infarction	92 (9.0)
Previous CABG	59 (5.8)
Previous coronary angioplasty	147 (14.4)
History of IHD/CAD	245 (23.9)
Previous congestive heart failure	20 (2.0)
History of TIA/CVA	32 (3.1)
Peripheral Arterial Disease	3 (0.3)
**Chief presenting complaint**	
Chest pain	913 (89.2)
Shoulder pain	6 (0.6)
Neck pain	2 (0.2)
Epigastric pain	23 (2.2)
Shortness of breath	29 (2.8)
Palpitations	31 (3.0)
Diaphoresis	5 (0.5)
Giddiness	9 (0.9)
Others	5 (0.5)
**30-day MACE**	
Yes	68 (6.6)
No	955 (93.4)
**1-year MACE**	
Yes	96 (9.4)
No	927 (90.6)

## Appendix

See Table A, Table B
